# *Clostridium butyricum* MIYAIRI 588-Induced Protectin D1 Has an Anti-inflammatory Effect on Antibiotic-Induced Intestinal Disorder

**DOI:** 10.3389/fmicb.2020.587725

**Published:** 2020-10-30

**Authors:** Tadashi Ariyoshi, Mao Hagihara, Shuhei Eguchi, Aiki Fukuda, Kenta Iwasaki, Kentaro Oka, Motomichi Takahashi, Yuka Yamagishi, Hiroshige Mikamo

**Affiliations:** ^1^Department of Clinical Infectious Diseases, Aichi Medical University Graduate School of Medicine, Nagakute, Japan; ^2^Miyarisan Pharmaceutical Co., Ltd., Saitama, Japan; ^3^Department of Molecular Epidemiology and Biomedical Sciences, Aichi Medical University, Nagakute, Japan; ^4^Departments of Kidney Disease and Transplant Immunology, Aichi Medical University, Nagakute, Japan

**Keywords:** *Clostridium butyricum*, lipid mediators, protectin D1, G-protein coupled receptor 120, interleukin (IL)-4

## Abstract

Metabolites are thought as the end products in cellular regulatory processes and their levels show the strongest relationships with the phenotype. Previously, we showed that the administration of *Clostridium butyricum* MIYAIRI 588 (CBM 588) upregulated protectin D1, an anti-inflammatory lipid metabolite, in colon tissue under antibiotic therapy. However, how CBM 588 induces protectin D1 expression and whether the metabolite has anti-inflammatory effects on antibiotic-induced inflammation are unclear. Therefore, here, we evaluated the effect of CBM 588 on lipid metabolism and protectin D1 in gut protection from antibiotic-induced intestinal disorders. In the CBM 588 treatment group, expression levels of genes encoding lipid receptors related to the conversion of DHA to protectin D1, such as polyunsaturated fatty acid (PUFA) receptors, G-protein coupled receptor 120 (GPR120), and 15-lipoxygenase (LOX), were increased in colon tissue. CD4^+^ cells producing interleukin (IL)-4, the main component of T helper type 2 (Th2) cells that can activate 15-LOX, also increased in CBM 588-treated groups even after clindamycin co-administration. In addition, similar to CBM 588, exogenously administered protectin D1 reduced inflammatory cytokines, while IL-10 and TGF-β1, works as anti-inflammatory cytokines, were increased. Our data revealed that CBM 588 activated 15-LOX to enhance protectin D1 production by increasing IL-4-producing CD4^+^ cell population in the intestinal tract. Additionally, CBM 588-induced protectin D1 clearly upregulated IL-10-producing CD4^+^ cells to control antibiotic-induced gut inflammation. We provide new insights into CBM 588-mediated lipid metabolism induction for the treatment of gut inflammatory diseases.

## Introduction

Metabolites are the end products of cellular metabolism. They have a strong correlation with phenotype, and levels of metabolites also provide more comprehensive understanding of biological system’s response to genetic changes and environmental changes. Gobbetti et al. (2017) revealed that bioactive lipid mediators of docosapentaenoic acid (DPA) and eicosapentaenoic acid (EPA) metabolites have the functional roles in intestinal inflammation. These metabolites administrations attenuate dextran sulfate sodium (DSS)-induced colitis in mice through decreased leukocyte—endothelial interaction and reduced granulocyte trafficking. Although the inflammatory bowel diseases (IBDs), Crohn’s disease and ulcerative colitis (UC) are characterized by chronic inflammation of the gastrointestinal (GI) tract of unknown etiology (Gajendran et al., 2018), our previous study revealed that gut immunological and metabolic alterations related to antibiotic-induced dysbiosis also enhance gut inflammation and epithelial damage in colon tissue (Hagihara et al., 2018).

To devise a treatment method for dysbiosis-induced gut inflammation, research on probiotics is actively conducted. Probiotics are live microorganisms promoted health benefits when consumed, generally by controlling the gut flora (Hill et al., 2014). In particular, the *Clostridium butyricum* MIYAIRI 588 strain (CBM 588) is a gram-positive obligate anaerobic bacterium that has been used to treat human gastrointestinal diseases in Japan (Seki et al., 2003). In our previous study, CBM 588 attenuated antibiotic-induced gut inflammation through immunological modulation. We also admitted the upregulation of anti-inflammatory lipid metabolites, such as palmitoleic acid, 15d-PGJ2, and protectin D1 in host colon tissue (Hagihara et al., 2020). However, the effects of CBM 588 administration on lipid metabolic changes and whether these alterations have a crucial role in the anti-inflammatory effects remain unclear.

Therefore, to elucidate the effects of CBM 588 and its mechanisms, we investigated lipid metabolism, receptor expression related to lipid mediators, and immunological effects under antibiotic-induced dysbiosis with CBM 588 administration. Consequently, this study is the first to reveal that CBM 588-induced protectin D1 production in colon tissue through the upregulation of interleukin (IL)-4 via G-protein coupled receptor (GPR) 84 or GPR 120. Additionally, CBM 588-induced protectin D1 production attenuated antibiotic-induced gut inflammation through upregulation of IL-10-producing CD4^+^ cells in colon lumina propria (cLP). These findings provide additional insights into the prevention and treatment of inflammation with CBM 588.

## Materials and Methods

### Reagents

CBM 588 powder provided by Miyarisan Pharmaceutical Co., Ltd. The powder consisted of 2.2 × 10^10^ colony forming unit (cfu)/g (Lot No. 61GT, Tokyo, Japan) of viable spores of CBM 588. The powder was used for all *in vivo* studies. Clindamycin (Dalacin^®^ S Injection; CLDM) was purchased from Pfizer Japan Inc. (Tokyo, Japan). Protectin D1 [10(S),17(S)-dihydroxy-4Z,7Z,11E,13Z,15E,19Z-docosahexaenoic acid] was purchased from Cayman U.S.A. Inc. (Ann Arbor, MI, United States). The 15-lipoxygenase (LOX) inhibitor (PD146176) was purchased from Cayman U.S.A. Inc. Immediately before each *in vivo* experiment, CBM 588 powder was weighed and reconstituted with phosphate-buffered saline (PBS, pH 7.4). CLDM, protectin D1, and 15-LOX inhibitor were further diluted in PBS to achieve the desired concentration. Both solutions were stored under refrigeration and discarded 12 h after reconstitution.

### Mouse Preparation and Housing

Specific-pathogen-free, female ICR mice (8–9 weeks old) were obtained from Charles River Laboratories Japan, Inc. (Yokohama, Japan) (body weight is approximately 30 g) and utilized throughout this experiment. The mice were maintained under the National Research Council recommendations. They were provided food and water *ad libitum*. The study was reviewed and approved by the Ethics Committee of Aichi Medical University (2018-75).

### Administration of Medicines

For lipid metabolism analysis, 12 female ICR mice were divided into four groups (*n* = 3): (I) control group, (II) CLDM administration group, (III) CBM 588 administration group, and (IV) combination group (CBM 588 + CLDM). CBM 588 spore powder was administered by oral gavage at 500 mg/kg/day (3.4 × 10^8^ cfu/g/mouse/day) of CBM 588. CLDM was also administered by oral gavage at 40 mg/kg/day. The CBM 588 spore powder was dissolved into PBS and then mixed. Mice were administered twice/day, at 10 a.m. and 4 p.m., using 1/2 doses every time, for 4 days and kept for another 4 days (Hagihara et al., 2020). For the other analyses, 25 mice were divided into five groups (*n* = 5): (I) control group, (II) CLDM administration group, (III) CBM 588 + CLDM combination group, (IV) protectin D1 + CLDM combination group, and (V) CBM 588 + CLDM + 15-LOX inhibitor combination group. Protectin D1 was administrated by intraperitoneal injection (IP) injection at 0.3 μg/100 μL in PBS/day and 15-LOX inhibitor was administrated by IP injection at 0.3 mg/300 μL in PBS/day (Gobbetti et al., 2017). CBM 588 powder and CLDM were administered as described above.

### Assessment of Physiological Conditions

Body weights reported as the percentage of weight loss from initial body weight. Colon length, colon weight, and cecum weight were measured at necropsy.

### Tissue Sample Preparation for Lipidomic Study

The colon tissue samples were homogenized in RIPA buffer (Nacalai Tesque, Kyoto, Japan). The suspension was centrifuged at 10,000 × *g* for 20 min at 4°C after sonication for 10 s. Frozen supernatants from mouse colon samples (stored at −80°C) were thawed on ice to 0°C. A portion of the supernatant sample (20 μL) was mixed with methanol (80 μL), immediately after thawing. The mixture was vigorously vortexed (1 min), and centrifuged (15,000 rpm, 3 min). The collected supernatant was dried under reduced pressure after mixing with acetonitrile (50 μL) and vortexing (1 min). The mixture was then centrifuged (15,000 × *g*, 3 min).

### Comprehensive Analysis of Lipid Mediators Using Liquid Chromatography/Mass Spectrometry (LC-MS/MS)

The orbitrap LC-MS/MS analyses were performed on a Vanquish H system (Thermo Fisher Scientific, Cleveland, OH, United States) using an Acclaim RSLC120 C18 (2.2 μm 2.1 i.d. × 150 mm, Thermo Fisher Scientific, Cleveland, OH, United States) and Q Exactive (Thermo Fisher Scientific, Cleveland, OH, United States) with an electrospray ionization device. The negative ion mode was used. Mobile phase A was consisted with MilliQ and 0.1% formic acid. Acetonitrile was used as mobile phase B. After equilibration with 20% mobile phase solution B, 2 μL of the sample was injected and measured. The flow condition of the LC was eluted at a flow rate of 0.4 mL/min. The linear gradient conditions, MS conditions, and ionization conditions are shown in Supplementary Tables 1–3. The raw files were produced by Xcalibur Version 4.1, using the software Compound Discoverer Version 3.1. Data (negative ion mode) were imported into the software to pre-process the data, filtering, alignment, and peak identification. The identified metabolites, retention times (Rt), mass-to-charge ratios (m/z), peak area values, MS and MS/MS fragments, and pathway information were obtained for all samples. The raw files were normalized by Compound Discoverer Version 3.1 software and MS and MS/MS fragments were used to compare the MS fragment and MS/MS fragment library mzCloud and MS fragment library Chem Spider. The metabolomics data generated during the current study were submitted to the EMBL-EBI MetaboLights database with the identifier^1^ MTBLS2087.

### RNA Isolation and cDNA Preparation

As previously described (Hagihara et al., 2020), total RNA was isolated by homogenizing mouse colons (30 mg) using an RNA isolation kit (MACHEREY-NAGEL, Düren, Germany) in accordance with the protocol manufactures provided. Subsequently, to prepare complementary DNA (cDNA), a high-capacity RNA to cDNA kit (Thermo Fisher Scientific, CA, United States) was used in accordance with the manufacturer’s instructions. Total RNA solution (9 μL) was mixed with 2 × RT Buffer mix and 20 × RT Enzyme Mix to be final volume of 20 μL. The mixture was incubated at 37°C (60 min), and stopped by heating to 95°C (5 min) and holding at 4°C. For convenience, the incubation was performed in a thermal cycler. The cDNA was either immediately used in real-time PCR applications or placed for long-term storage in a freezer (−20°C).

### Quantitative Real-Time Polymerase Chain Reaction (RT-PCR)

The PowerUp^TM^ SYBR Green PCR Master Mix (Applied Biosystems, United States) was used to conduct quantitative RT-PCR according to the manufacturer’s protocol. PCR reactions were performed with reaction mixture containing of cDNA (2 μL), PowerUp^TM^ SYBR Green PCR Master Mix (5 μL), 10 μM forward primer (0.5 μL), 10 μM reverse primer (0.5 μL), and nuclease-free water (2 μL) (total volume: 10 μL). The primer sequences are shown in Supplementary Table 4. The following RT-PCR protocol was used, (i) Tm ≥ 60°C: 50°C (2 min), 95°C (2 min), and 40 cycles of denaturation at 95°C (15 s) with annealing/extension at 60°C (1 min), (ii) Tm < 60°C: 50°C (2 min), 95°C (2 min), with 40 cycles of denaturation at 95°C (15 s), annealing at 60°C (15 s), and extension at 72°C (1 min). For relative quantitation, we used β-actin as an endogenous reference to compare the amount of normalized target and used ΔΔCt method to analyze data. Each relative RNA expression level was normalized to the control group (represented as RQ).

### Protein Expressions Related to Lipid Metabolism

The colon tissue samples were homogenized in RIPA buffer (Nacalai Tesque, Kyoto, Japan). After sonication (10 s), the suspension was centrifuged (10,000 × g, 20 min) at 4°C. Protein expression levels of the lipid metabolism related proteins, GPR120 and 15-LOX in the supernatants were measured with commercially available mouse enzyme-linked immunosorbent assay (ELISA) kits (Biolegend, CA, United States). The procedures were performed according to the manufacturer’s instructions.

### Histopathological Evaluation

The colon was fixed with 10% neutral-buffered formalin for 48 h. After embedded in a paraffin block, the sections of the sample were measure the severity of inflammation after hematoxylin and eosin (H&E) staining. Pathological expert performed histologic scoring using a following criteria [the rare inflammatory cells in the lamina propria (counted as 0), increased numbers of inflammatory cells in the lamina propria (counted as 1), confluence of inflammatory cells extending into the submucosa (counted as 2), and transmural extension of the infiltrate (counted as 3)]. For the scoring of tissue damage, the pathologist counted as 0 when no mucosal damage was admitted, 1 when discrete lymphoepithelial lesions were admitted, 2 when surface mucosal erosion was admitted, and 3 when extensive mucosal damage and extension through deeper structures of the bowel wall were admitted. The combined histologic score ranged from 0 (no changes) to 6 (extensive cell infiltration and tissue damage) (Hagihara et al., 2020).

### Assessment of Oxidative Stress

The colon tissue samples were homogenized in RIPA buffer (Nacalai Tesque, Kyoto, Japan). After sonication (10 s), the suspension was centrifuged (10,000 × *g* for 20 min) at 4°C. Protein expression levels of superoxide dismutase (SOD), total glutathione (DOJINDO, Kumamoto, Japan), malondialdehyde (MDA) (abcam, Cambridge, United Kingdom) and total antioxidant capacity (TAC) (Biovision, CA, United States) were also measured using assay kits to reveal the oxidative stress.

### Cytokine Assessment

Sample preparation was done as in the oxidative stress assessment. Protein expression of IL-1β, tumor necrosis factor-α (TNF-α), transforming growth factor-β1 (TGF-β1), IL-10, and IL-4 in the supernatants were measured with ELISA kits (Biolegend, CA, United States). The procedures were performed in accordance with the manufacturer’s instructions.

### Isolation of Lymphocytes From the Colon Tissue

To isolate colon lumina propria lymphocytes (cLP), collected large intestines were opened longitudinally and washed to remove fecal content. After that, the sample was shaken in HBSS containing 1.5% fetal bovine serum (FBS) and 0.5 M EDTA (20 min) at 37°C. After removing epithelial cells and fat tissue, the intestines were cut into small pieces and incubated with HBSS containing 1.5% FBS and 1 mg/mL collagenase type IV (CLSS, WOR, NJ, United States) (1 h) at 37°C in a shaking water bath. The digested tissues were processed through a 100 μm filter (pluriSelect, Leipzig, Germany) and suspended in 5 mL of 40% Percoll (GE Healthcare, Tokyo, Japan) before overlay on 5 mL of 80% Percoll in a 15 mL Falcon tube. Percoll gradient separation was performed by centrifugation at 800 × *g* for 20 min at 20°C. The interface cells were collected and used as cLP lymphocytes.

### Evaluation of the Inhibition of 15-LOX in Mouse Colon cLP

To investigate the involvement of 15-LOX in the production of protectin D1 by CBM 588, cLP were cultured with 15-LOX inhibitors (or vehicle) for 24?h. To selectively inhibit the target enzymes, the PD146176 concentrations were reported (Pistorius et al., 2018). After incubation, the culture was suspended and stored at −80°C, and subsequently assayed for cytokines.

### Flow Cytometric Analysis

The collected cells were suspended in RPMI 1640 containing 10% FBS. For detection of IL-10 and IL-4, lymphocytes were stimulated for 4 h with PMA (Sigma, St. Louis, MO, United States) at 50 ng/mL and ionomycin (Sigma) at 1 μg/mL. BD Cytofix/Cytoperm kit (554714, BD Biosciences, Franklin Lakes, NJ, United States) was used to fix and permeabilize after first stain for surface CD3 and CD4. After that the cells were stained for intracellular IL-10 and IL-4. The following antibodies were used: FITC-labeled anti-CD4 cell (561535, BD Biosciences), Per-CP-labeled anti-CD3 Ab (561089, BD Biosciences), APC-labeled anti-IL-10 Ab (554468, BD Biosciences), and PE-labeled anti-IL-4 Ab (8184629, BD Biosciences). Flow cytometry was performed using FACSCanto^TM^ II (BD Biosciences). FlowJo software (TreeStar Inc., Williamson Way Ashland, OR, United States) was used to analyzed the data.

### Statistics and Analysis

For quantitative data, all results are shown as the mean ± *SD* (*n* = 3–5). One-way ANOVA with Tukey’s test was applied to compare LC-MS/MS signal intensity, body weight, colon length, colon weight, cecum weight, colon pathology score, gene expression level (RT-PCR), protein expression level (ELISA), and percentage of IL-4- and IL-10-positive cells by flow cytometric analysis. A probability of *p* < 0.05 was considered statistically significant. ^∗^*p* < 0.05 compared with control. ^∗∗^*p* < 0.01 compared with control (except Figure 7B). Statistical analysis of the multiple group comparisons was performed using a one-way analysis of variance followed by Tukey’s test; ^∗^*p* < 0.05 compared with CLDM + CBM588 (Figure 7B).

## Results

### *C. butyricum* Administration Changes Lipid Metabolism in Host Colon Tissue, Even Under Antibiotic Administration

**FIGURE 1:**
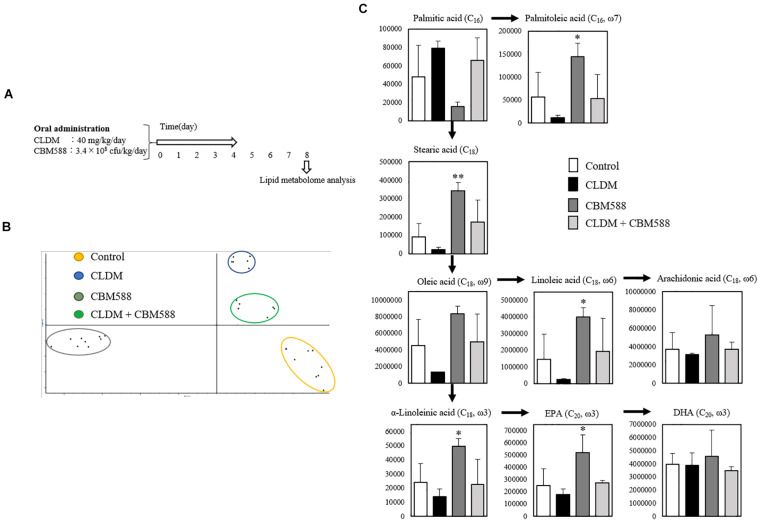
Effect of clindamycin (CLDM) and *Clostridium butyricum* MIYAIRI 588 strain (CBM 588) on lipid metabolism in mouse colon. **(A)** Experimental design of *in vivo* study with 9-week-old ICR mice. Mice were divided into groups as follows and received CLDM and/or CBM 588 via sonde for 4 days: control group (Control), CLDM administration group (CLDM), CBM 588 administration group (CBM 588), and combination group (CLDM + CBM588). Colon tissue was harvested on day 8 for lipid metabolome analysis. **(B)** PCA of major sources of lipid metabolite variability in the colon. Data points indicate colon samples from three independent experiments (biological replicates; *n* = 3) injected randomly into the LC-MS/MS system. **(C)** Metabolic pathways of palmitic acid conversion to arachidonic acid (AA), eicosapentaenoic acid (EPA), and DHA. The vertical axis indicates signal intensity. Number of carbon atoms and double bond position are shown in parentheses (*n* = 3). *p < 0.05 and **p < 0.01 compared with control.

We previously confirmed that colonization of CLDM-sensitive CBM 588 was maintained in the colon after CLDM administration (Hagihara et al., 2020). To reveal the impacts of gut colonization by CBM 588 on lipid metabolism in regulating gut homeostasis, we conducted *in vivo* study (Figure 1A), and analyzed lipid metabolites in mouse colon by LC-MS/MS. Consequently, a total of 176 fatty acid metabolites were identified. As samples clustered in principal component analysis (PCA) according to the treatments, CLDM and CBM 588 administration affected gut lipid metabolism (*p* < 0.05) (Figure 1B). A heatmap was created by hierarchical Pearson clustering using compounds that have twice or half the signal area of the control, as shown in Supplementary Figure 1. The results showed a significant difference between each group for a total of 40 significant metabolites (*p* < 0.05). In particular, palmitoleic acid, stearic acid, oleic acid, linoleic acid, α-linolenic acid, and EPA were significantly increased with CBM 588 administration than that of control group (Figure 1C). Palmitoleic acid (C_16_, omega-7), is classified as a lipokine, is a monounsaturated fatty acid of palmitic acid (C_16_). Lipokine is known as the metabolites can suppress inflammatory cytokine production. Additionally, it attenuates inflammation in metabolically active tissues (Cao et al., 2008; Chan et al., 2015). Additionally, CBM 588 administration enhanced stearic acid (C_18_) and oleic acid (C_18_, omega-9) production (Figure 1C), which were metabolized to docosahexaenoic acid (DHA) and EPA. Among lipid metabolites, lipokine, EPA, and DHA are metabolized to an anti-inflammatory lipid mediator (Kuda et al., 2018). Hence, we hypothesized that CBM 588 did not only regulate the production of other pro-inflammatory lipid metabolites, but also enhanced the production of other anti-inflammatory lipid metabolites in colon tissue. However, the mechanism by which lipid metabolic changes induced by CBM 588 protect the colon from antibiotic damage remains to be clear. Therefore, we investigated detailed lipid metabolic profile alterations, receptor expression, and cytokine profiles in isolated colon tissue.

### *C. butyricum* Negatively Modulates Arachidonic Acid-Derived Pro-inflammatory Lipid Metabolites and Upregulates DHA–EPA-Derived Anti-inflammatory Lipid Metabolites

**FIGURE 2:**
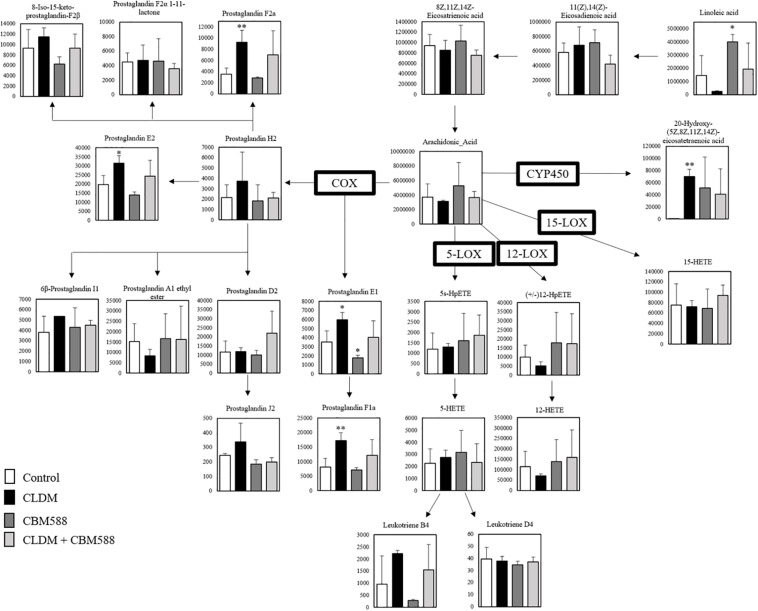
Clindamycin (CLDM) induces prostaglandin production from linoleic acid via arachidonic acid (AA). The metabolic pathway is centered on AA. The signal intensities of the metabolites were compared using the average value of each group. The vertical axis indicates the signal intensity. Enzymes used for the metabolism of each lipid are indicated in bold (*n* = 3). HpETE, Hydroperoxyeicosatetraenoic acid; HETE, Hydroxyeicosatetraenoic acid; COX, Cyclooxygenase; LOX, Lipoxygenase; CYP450, Cytochrome P450. *p < 0.05 and **p < 0.01 compared with control.

**FIGURE 3:**
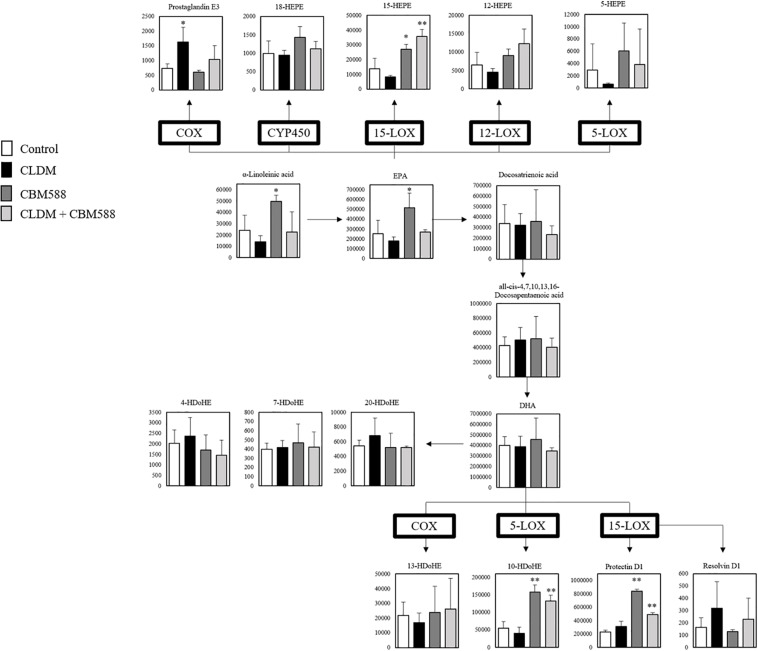
*Clostridium butyricum* MIYAIRI 588 strain (CBM 588) induces protectin D1 production from α-linoleinic acid via eicosapentaenoic acid (EPA) and DHA. The metabolic pathway for the production of protectin D1. The signal intensities of the metabolites were compared using the average value of each group. The vertical axis indicates the signal intensity. Enzymes used for the metabolism of each lipid are indicated in bold (*n* = 5). HEPE, hydroxyeicosapentaenoic acid; HDoHE, hydroxydocosahexaenoic acid; COX, cyclooxygenase; LOX, lipoxygenase. *p < 0.05 and **p <0.01 compared with control.

Next, we focused on the arachidonic acid (AA) metabolic cascade (Figure 2) and EPA-DHA metabolic cascade (Figure 3). AA is one of the sources of omega-6 PUFAs and is a substrate for the synthesis of a wide variety of eicosanoids, and is known to be pro-inflammatory, vasoconstrictive, and/or pro-aggregatory (Larsson et al., 2004; Harris et al., 2009; Innes and Calder, 2018). Consequently, in our study, prostaglandins (PG) F1a, F2a, E1, and E2 were significantly in the CLDM administration group (Figure 2). Reduced AA levels (the biosynthetic precursor of PGF2α), 19H-PGF1α (the metabolite of prostacyclin), and 20H-PGF2α (the metabolite of PGF2α), suggest the attenuation of gut inflammation *in a vivo* study with a DSS model (Lee et al., 2017). Additionally, PGE2 is a major inflammatory mediator (Kawahara et al., 2015). Meanwhile, contrary to AA, EPA and DHA, are classified as omega-3 PUFAs and are known to be inflammatory cytokine suppressors (Serhan et al., 2008; Calder, 2015). Our *in vivo* study showed that 15- hydroxyeicosapentaenoic acid (HEPE), 10 HDoHE, and protectin D1 in the omega-3 PUFA cascade were significantly higher in the CBM 588 administration group and the combination group (Figure 3). EPA-derived 11-HEPE, 12-HEPE, and 15-HEPE are associated with anti-inflammatory properties (Coras et al., 2019), and DHA-derived protectin D1, which have been identified as specialized pro-resolving lipid mediators (SPMs) important in intestinal protection, is a potent anti-inflammatory lipid mediator (Gobbetti et al., 2017). Collectively, these results indicate that CLDM administration induces pro-inflammatory AA metabolism, whereas CBM 588 administration preferentially induces anti-inflammatory DHA and EPA metabolism.

### *C. butyricum* Administration Induced the Upregulation of Protectin D1 Through 15-LOX Upregulation

**FIGURE 4:**
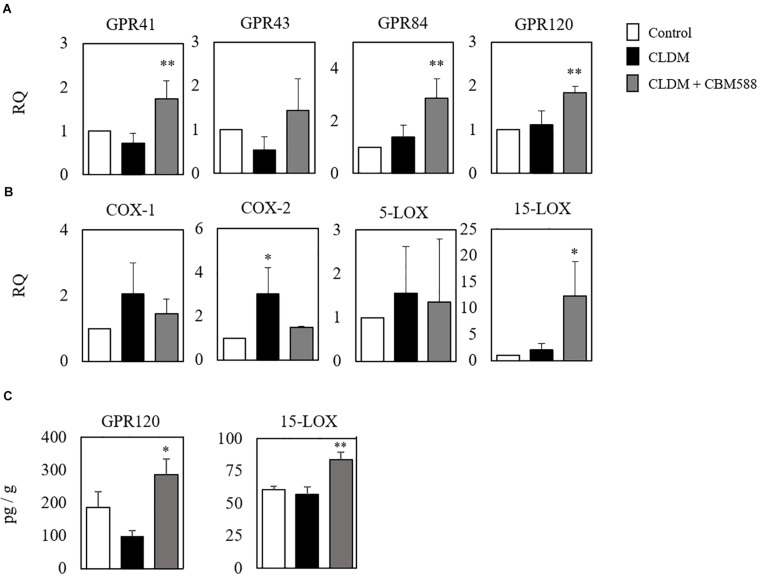
*Clostridium butyricum* MIYAIRI 588 strain (CBM 588) induces the up-regulation of fatty acid receptors and 15-lipoxygenase gene expression. **(A)** qPCR results showing the relative RNA levels of genes encoding G-protein-coupled receptor (GPR) that recognizes fatty acids in colon tissue of mice. **(B)** qPCR results showing the relative RNA levels of genes encoding enzymes related to lipid metabolism in colon tissue of mice. **(C)** ELISA results showing concentrations of GPR41, GPR120, COX, and 15-LOX in colon tissues of mice. Relative RNA levels of each target gene were normalized to those in the control group (represented as RQ in the vertical axis) (*n* = 5). *p < 0.05 and **p < 0.01 compared with control.

Although CBM 588 administration upregulated anti-inflammatory lipid metabolites production in colon tissue, metabolites mainly related to the anti-inflammatory effects of antibiotic-induced gut inflammation remain to be solved. To address this, the gene expression level related to fatty acid metabolism in colon tissue was evaluated by RT-PCR, and we found that CBM 588 administration increased several orphan GPRs RNA expression levels in colon tissue, such as of GPR41, GPR43, GPR84, and GPR120 (Figure 4A). They have been identified as receptors for free fatty acids. Since these receptors are classified by the length of the fatty acid carbon chain, medium-chain and long-chain fatty acids activate GPR40 and GPR120, short-chain fatty acids (SCFAs) activate GPR41 and GPR43, medium-chain fatty acids activate GPR84 (Hara et al., 2013; Miyamoto et al., 2017). Hence, our results suggest that CBM 588 administration activates the utilization of some fatty acids in colon tissue. In addition, cyclooxygenase (COX) is the enzyme that catalyzes the conversion of AA to PG endoperoxides (Vitale et al., 2016). The expression levels of COX-1 and COX-2 were upregulated in the CLDM-treated group (Figure 4B), while 15-LOX was significantly higher in the CBM 588-administered group (Figure 4B). From DHA, protectin D1 is formed via the 15-LOX pathway (Serhan et al., 2015). Of note, the protein expression levels of GPR120 and 15-LOX were significantly higher in the CBM 588-administered group (Figure 4C). Taken together, these results suggest that antibiotics and CBM 588 administration affected the expression of some genes and proteins related to fatty acid metabolism. Notably, our results revealed that CLDM administration induced the upregulation of COX-1 and COX-2 to enhance AA metabolism and produce pro-inflammatory lipid metabolites. In addition, CBM 588 administration induced GPR expression in colon tissue to facilitate 15-LOX expression. These results support the fact that CBM 588 administration led to the upregulation of protectin D1 and resulted in anti-inflammatory effects.

### Protectin D1 Prevents Antibiotic-Induced Colon Tissue Inflammation

**FIGURE 5:**
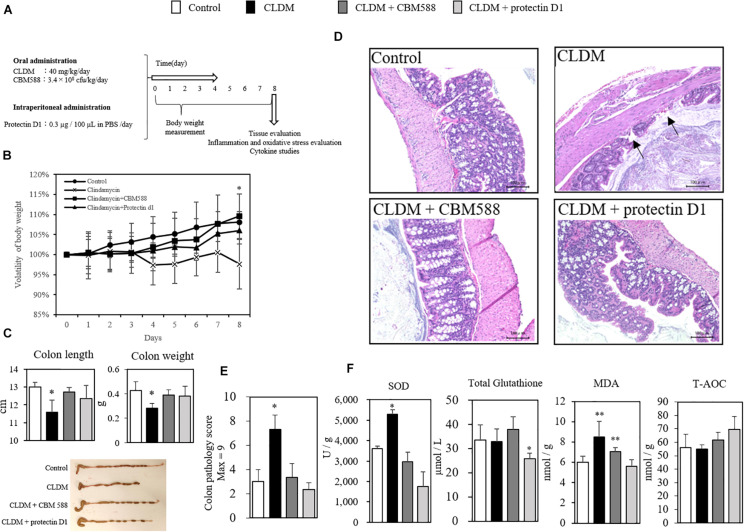
*Clostridium butyricum* MIYAIRI 588 strain (CBM 588) and protectin D1 suppress inflammation in antibiotic-induced colitis. **(A)** Experimental design of *in vivo* study with 9-week-old ICR mice. Mice were divided into groups as follows and received CLDM and/or CBM 588 via sonde and/or protectin D1 via intraperitoneal administration for 4 days: control group (Control), CLDM administration group (CLDM), CLDM + CBM 588 group (CLDM + CBM588), and CLDM + protectin D1 group (CLDM + protectin D1). Colon tissue was harvested on day 8 for tissue evaluation, inflammation and oxidative stress marker measurement, cytokine studies, and comprehensive metabolome analysis. **(B)** Time course showing weight changes of control group (∙), CLDM administration group (× ), CLDM + CBM 588 group (■) and CLDM + protectin D1 group (▲), with representative data for *n* = 5 per group. The results are represented as mean ± *SD*. Statistical analysis of the multiple group comparisons was performed using a one-way analysis of variance followed by Tukey’s test; ^∗^*p* < 0.05 compared with control. **(C)** Colon length (cm) and weight (g), with representative data *n* = 5 per group, mean ± *SD*. Pictures show representative isolated colons from each group (*n* = 5). **(D)** Colons revealed superficial epithelial necrosis and the presence of inflammatory cells only in the CLDM group with hematoxylin and eosin (H&E) staining (magnification, 20 × ; scale bar, 100 μm). **(E)** Histopathological scoring of H&E-stained colon sections in each group (*n* = 5). **(F)** Protein concentrations of oxidative stress markers in colon tissues of mice (*n* = 5). *p < 0.05 and **p < 0.01 compared with control.

We found that CBM 588 administration induced the protectin D1 production through the upregulation of 15-LOX expression in colon tissue. However, the impact of protectin D1 upregulation in colon tissue under antibiotic-induced gut inflammation remains unclear. To determine whether protectin D1 induced by CBM 588 in gut has an immunomodulatory impact to regulate gut homeostasis, we conducted *in vivo* study with CLDM, CBM 588 and protectin D1 (Figure 5A). Consequently, we confirmed that protectin D1 administration prevented weight loss due to CLDM administration (Figure 5B). Similar to the colitis model induced by DSS that shows shortened colon length (Hayashi et al., 2013; Wang et al., 2017), we found a shortened colon length only in the CLDM-administered group, while the combination group and CLDM + protectin D1 administration group showed prevention of shortening of the colon length (*p* < 0.05) (Figure 5C). Upon histological examination on day 8, only the CLDM administration resulted in superficial epithelial necrosis and migration of inflammatory cells (Figure 5D). On the other hands, both CBM 588 and CLDM + protectin D1 administration group retained histologically normal colon mucosa, when CLDM was administered concurrently. CLDM administration group had significantly more epithelial damage in the colon, and the histological inflammation score of CLDM administration group was higher than that of the control and CBM 588 groups (Figure 5E). Furthermore, SOD and MDA were significantly increased in the CLDM mono-administered group. In contrast, CBM 588-administered group and the protectin D1-administered group were not different from control group. The total amount of glutathione was significantly decreased in the protectin D1 combined administration group. Conversely, total antioxidant capacity (T-AOC) increased in the protectin D1-administered group, while no significant difference was confirmed (Figure 5F). An inflammatory stimulus result in the initiation of carcinogenesis, which is mediated by reactive oxygen species (ROS) that may be direct, or mediated by the signaling pathways activated by ROS (Reuter et al., 2010). Collectively, our results indicate that protectin D1 administration attenuated antibiotic-induced gut inflammation, and further suggest that protectin D1 shows anti-oxidant effects.

### Protectin D1 Downregulate Pro-inflammatory Cytokine Expressions in Antibiotic-Induced Dysbiosis

**FIGURE 6:**
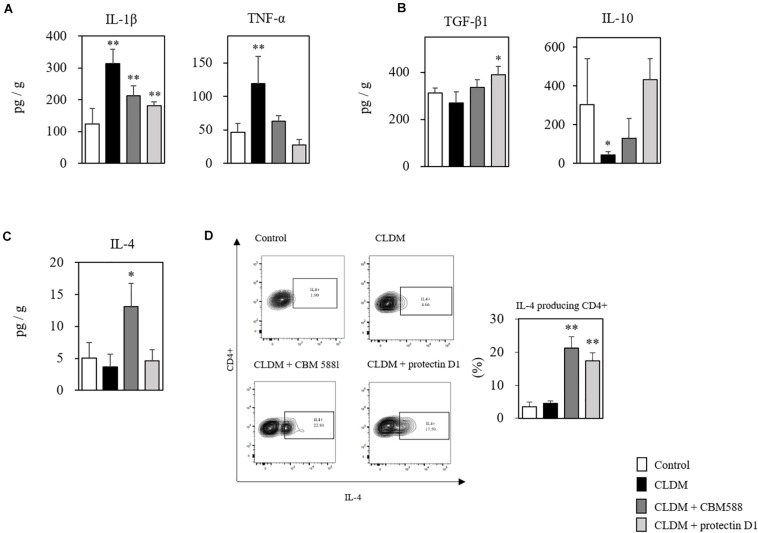
*Clostridium butyricum* MIYAIRI 588 strain (CBM 588) and protectin D1 modulates cytokines in antibiotic-induced colitis. **(A)** ELISA results showing concentrations of inflammatory cytokines in colon tissues of mice. **(B)** ELISA results showing concentrations of anti-inflammatory cytokines in colon tissues of mice. **(C)** ELISA results showing concentrations of cytokines up-regulated in colon tissues of mice following CBM 588 or protectin D1 administration (*n* = 5). **(D)** Representative flow cytometry plots of IL-4-producing CD4^+^ cells and percentage of IL-4-producing CD4^+^ cells in lymphocytes of colon lumina propria (cLP) (*n* = 3). *p < 0.05 and **p < 0.01 compared with control.

Next, to elucidate the immunological impacts of protectin D1 on gut epithelial protection, we assessed cytokine profiles of isolated colon tissues with RT-PCR and ELISA. CBM 588 has a colitis-preventing effect (Hayashi et al., 2013). We also found that various pro-inflammatory cytokines, such as IL-1β and TNF-α, were increased in the non-control group due to the effect of CLDM administration, but increased most in the CLDM mono-administered group (Figure 6A). Anti-inflammatory cytokine, such as TGF-β1 and IL-10, were significantly increased in the CBM 588- and protectin-D1 co-administered groups compared with CLDM mono-administered group (Figure 6B). CBM 588 upregulate regulatory T cell (Treg) generation via TGF-β_1_ induced by dendritic cells (DCs) in a DSS-induced acute colitis model (Kashiwagi et al., 2015). The TGF-β_1_ pathway promotes IL-10 cytokine production, while the pathway suppresses the productions of inflammatory cytokines (Hayashi et al., 2013; Huang et al., 2013). Hence, our data suggest that protectin D1 showed anti-inflammatory effect through a TGF-β_1_-dependent reduction of inflammatory cytokine expression and the upregulation of IL-10 cytokine. Interestingly, IL-4 was significantly increased only in the CBM 588 administration group in colon tissue (Figure 6C), and CBM 588 administration group showed a higher occupancy of IL-4-producing CD4^+^ cells than that in the protectin D1 administration group (Figure 6D). IL-4 promotes the differentiation of naive CD4^+^ T cells into CD4^+^ Th2 effector cells (Chen et al., 2004), and Th2 cells activate 15-LOX (Ariel et al., 2005). Taken together, CBM 588 and protectin D1 attenuated colon tissue inflammation through pro- and anti-inflammatory cytokine modulations. Under CLDM-induced gut inflammation, TGF-β_1_ and IL-10 can play an important role in the anti-inflammatory effects of protectin D1 administration. Additionally, as the protectin D1 co-administration group showed lower IL-4 levels than the CBM 588 administration group, we speculate that IL-4 can accelerate CBM 588-induced protectin D1 upregulation.

### *C. butyricum*-Induced Protectin D1 Enhances IL-10 Production in Colon Tissue

**FIGURE 7:**
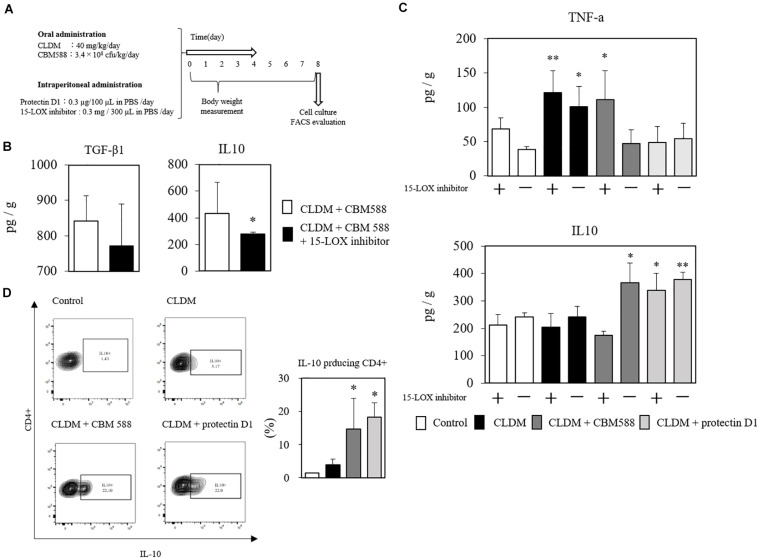
*Clostridium butyricum* MIYAIRI 588 strain (CBM 588)-induced protectin D1 enhance IL-10 production in colon tissue. **(A)** Experimental design of clindamycin (CLDM), CBM 588, and protectin D1 administration in 9-week-old ICR mice. Mice were divided into groups as follows and received CLDM and/or CBM 588 via sonde and/or protectin D1 via intraperitoneal administration for 4 days: control group (Control), CLDM administration group (CLDM), CLDM + CBM 588 group (CLDM + CBM588), CLDM + protectin D1 group (CLDM + protectin D1), and CLDM + CBM 588 + 15-LOX inhibitor group (CLDM + CBM588 + 15-LOX inhibitor). Colon tissue was harvested on day 8. **(B)** Result of *in vivo* 15-LOX inhibitor evaluation. Colon tissues were harvested for detection of anti-inflammatory cytokines (*n* = 5). Statistical analysis of the multiple group comparisons was performed using a one-way analysis of variance followed by Tukey’s test; ^∗^*p* < 0.05 compared with CLDM + CBM588. **(C)** Result of *in vitro* 15-LOX inhibitor evaluation. (i) Treatment with 15-LOX inhibitor (*n* = 3). **(D)** Representative flow cytometry plots of IL-10-producing CD4^+^ cells, and percentage of IL-10-producing CD4^+^ cells in lymphocytes of colon lumina propria (cLP) (*n* = 3). *p < 0.05 and **p < 0.01 compared with control.

IL-4 can accelerate protectin D1 production through 15-LOX upregulation in monocytes in colon tissue (Mukherjee et al., 2004; Ariel et al., 2005; Calandria et al., 2009). To reveal that the protective effects of protectin D1 induced by CBM 588 were associated with the upregulation of 15-LOX, we used an anti-15-LOX antibody to the combination (CLDM + CBM 588 administration) group, and compared it with the CLDM + protectin D1 administration group (Figure 7A). Consequently, CBM 588 did not ameliorate the gut inflammation induced by CLDM when used with an anti-15-LOX antibody. At the same time, TGF-β_1_ and IL-10 were downregulated by the anti-15-LOX inhibitor in CBM 588-treated mice (Figure 7B). We further isolated the lymphocytes in cLP and found IL-10 reduction with the 15-LOX inhibitor, while TNF-α was found to be increased (Figure 7C). Notably, an upregulation of IL-10-producing CD4^+^ cells was observed in the combination group and the protectin D1 administration group (Figure 7D), compared with that in the CLDM administration group (*p* < 0.05). Collectively, our results revealed that 15-LOX accelerated the upregulation of CBM 588-induced protectin D1 upregulation. Additionally, IL-10-producing CD4^+^ cells induced by protectin D1 play an important role in protecting the colon from inflammation damage.

## Discussion

Our previous study revealed that CBM 588 administration improved mucosal damage and inflammation by dysbiosis of administration of CLDM, then *Bifidobacterium spp., Lactobacillus spp., Lactococcus spp*., were predominantly changed. Furthermore, CBM 588 administration promoted anti-inflammatory lipid metabolites productions through gut metabolite alterations via gut microbiome modulation (Hagihara et al., 2018, 2020). However, the identity of these CBM 588-induced metabolites and whether they show anti-inflammatory effects have not been entirely clear. Consequently, the antibiotic-induced lipid metabolic alterations were confirmed by exhaustive metabolomics in the current *in vivo* study. CBM 588 administration also had some effects on lipid metabolism, even under CLDM administration (Figure 3). In the CLDM administration group, PGE1, PGE2, PGF1, and PGF2, in the AA cascade were significantly induced (Figure 2) through COX-1 and COX-2 upregulation in the CLDM administration group (Figure 4B). In the *in vivo* colitis model, AA release and subsequent prostanoids, such as PGE1, PGE2, and PGF1 were significantly increased in the colonic mucosa (Hoang et al., 2007). PGE1 and PGF2 are produced in large amounts in cultures of IBD cells (Kusugami et al., 1991). Additionally, PGE2 levels are increased according to the severity of inflammation (Almeida et al., 2019). Hence, these results suggested that CLDM administration induced pro-inflammatory lipid mediators through COX enzyme upregulation. However, we did not observe this pro-inflammatory metabolite upregulation in the CBM 588 administration group even under CLDM co-administration. Hence, we speculated that co-administration of CBM 588 would induce EPA or DHA derived anti-inflammatory lipid mediators even under CLDM administration. In particular, EPA and DHA are metabolized to anti-inflammatory lipid mediators, while AA is metabolized to a pro-inflammatory lipid mediator (Kuda et al., 2018).

Consequently, palmitoleic acid, which suppresses pro-inflammatory cytokine production and macrophage polarization via the AMP-activated protein kinase activation in metabolically active tissues (Cao et al., 2008; Chan et al., 2015), was upregulated in the CBM 588 administration groups (Figure 3). EPA-derived 15HEPE and 12HEPE have anti-inflammatory properties and play an important role in the resolution phase of inflammation (Miller et al., 1990; Schacky et al., 1990; Zulfakar et al., 2007); these were also upregulated following CBM 588 administration (Figure 3). Hence, these anti-inflammatory lipid metabolites cause inflammatory cytokine suppression and induce anti-inflammatory cytokines. Contrastingly, PGE3, which is generally classified as a pro-inflammatory marker, was also increased in the CLDM administration group than in the CBM 588 administration group. However, PGE3 increased paracellular permeability to the same extent as PGE2 and redistribution of tight junction proteins (Rodríguez-Lagunas et al., 2013). Moreover, in this study, CBM 588 promoted protectin D1 production in colon tissue in colon tissue under an antibiotic-induced colitis (Figure 3). In the EPA and DHA metabolic cascade, protectin D1 is a member of the class of SPMs and possesses strong anti-inflammatory activity (Gobbetti et al., 2017). Protectin D1 can potentially reduce inflammation through the attenuation of oxidative stress and the inhibition of the pro-apoptotic signal, thereby preventing cellular degeneration (Mukherjee et al., 2004; Calandria et al., 2009). Hence, these findings indicate that CBM 588 has the potential to become a useful species for the prevention and treatment of gut inflammatory diseases due to intestinal barrier dysfunction-related diseases.

To reveal the impact and mechanisms of CBM 588 under antibiotic-induced gut inflammation, among pro-inflammatory lipid mediators induced by CBM 588, we focused on protectin D1 because it has potent anti-inflammatory effects on colitis (Gobbetti et al., 2017). Additionally, gene expression data showed GPR 84, GPR120, and 15-LOX RNA upregulation (Figure 4A), supporting our speculation that protectin D1 plays an important role in anti-inflammatory effects under antibiotic-induced gut inflammation. Protectin D1 is produced as a result of Th2-mediated activation of 15-LOX stimulated by IL-4 (Mukherjee et al., 2004; Ariel et al., 2005; Calandria et al., 2009). In relation to IL-4 production, GPR84 and GPR120, which recognize medium-chain and long-chain fatty acids, respectively, have been reported to be important for increasing IL-4 expression in activated T cells (Venkataraman and Kuo, 2005; Konno et al., 2016). Additionally, we observed potent IL-4 upregulation in the CBM 588 administration group in colon tissue and cLP, compared with the protectin D1 administration group (Figures 6C,D). Hence, we thought that CBM 588 administration facilitates IL-4 production via GPR84 and GPR120 signaling and induces protectin D1 production. As supportive data, 15-LOX was upregulated with CBM 588 administration compared to that in the other groups (Figure 4B). Hence, these results revealed that CBM 588-induced IL-4 secretion facilitated protectin D1 production via 15-LOX upregulation.

The current study focused on only protectin D1, although we also observed other anti-inflammatory lipid mediators, such as palmitoleic acid, EPA-derived 15HEPE, and 12HEPE. However, the 15-LOX inhibitor attenuated the anti-inflammatory effects of CBM 588 (Figures 7B,C). Hence, it is clear that CBM 588-induced protectin D1 has an important role to show anti-inflammatory effects under antibiotic-induced gut inflammation. Additionally, similar to the CBM 588 administration group, protectin D1 administration upregulated IL-10 production in colon tissue through IL-10-producing CD4^+^ cell upregulation (Figures 6B, 7D). IL-10, which is classified as an anti-inflammatory cytokine, is produced by some types of monocytes, such as Th2 cells (IL-4-producing CD4^+^ cells), mast cells, CD4^+^CD25^+^Foxp3^+^ Tregs in the intestine (Hayashi et al., 2013; Fang and Zhu, 2020). Additionally, CBM 588 induces intestinal IL-10-producing cells to suppress acute experimental DSS-induced colitis in mice (Hayashi et al., 2013). Hence, CBM 588-induced protectin D1 shows anti-inflammatory effects through IL-10-producing CD4^+^ cell upregulation.

**FIGURE 8:**
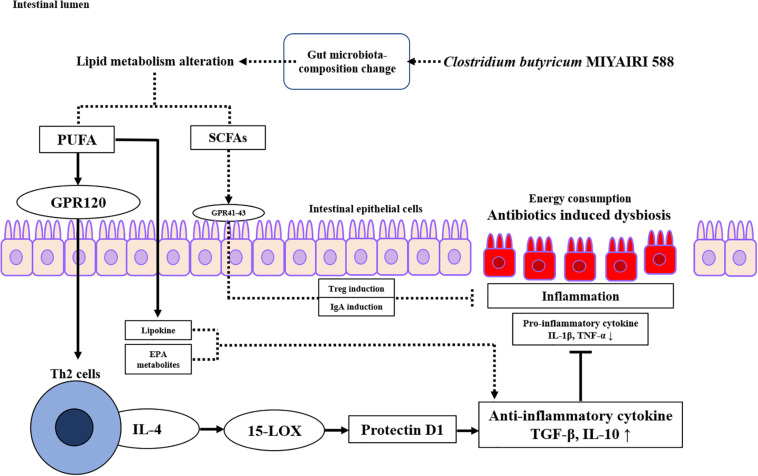
Proposed signaling pathway involved in protectin D1 expression induced by *Clostridium butyricum* MIYAIRI 588 strain (CBM 588) against antibiotic-induced inflammation. CBM 588 stimulated poly-unsaturated fatty acid (PUFA) metabolism, and PUFAs served as ligands of G-protein-coupled receptor 120 (GPR120) on Th2 cells. It was speculated that the stimulated Th2 cells produced IL-4 and activated 15-LOX, resulting in the induction of protectin D1. In addition, protectin D1 induced anti-inflammatory cytokines to control antibiotic-induced gut inflammation through IL-10 upregulation. Solid arrows indicate the results of the present study.

Our study revealed some new mechanistic insights into how CBM 588 modulates anti-inflammatory effects via lipid metabolism alternations against antibiotic-induced gut inflammation (Figure 8). However, there are limitations. The first, the mouse gut microbiome is not identical to the human gut microbiome (Rosshart et al., 2019). Hence, further research is needed to investigate whether these results can be reproduced in humans. The second, the number of samples for the analysis of lipid metabolism was statistically small. However, mice used in this study were under same conditions, there was no significant difference in metabolites within each group, and the PCA analysis revealed that sample data were clearly separated in each group. Hence, the tendency of metabolites analysis was sufficiently characterized for each group, even if the number of samples is small. Finally, the female ICR mice of 8–9 weeks have become sexually mature, and the secretion of estrogen may affect the results of the test (Meng et al., 2020). However, all mice in each group in this study were under same conditions to minimize some impacts derive from physiological cycles. Additionally, we used same protocol (and female ICR mice) with previous works to reveal gut microbiome and short chain fatty acids concentrations in fecal samples, etc. (Hagihara et al., 2020). Hence, we used same ICR mice to align study condition with previous our study.

In summary, our study revealed that CBM 588 administration changed lipid metabolism in colon tissue, even in antibiotic-induced colitis. We found that CBM 588 enhanced EPA-DHA-derived protectin D1 through 15-LOX activation via IL-4-producing CD4^+^ cell upregulation. Additionally, CBM 588-induced protectin D1 showed anti-inflammatory effects through the IL-10-producing CD4^+^ cell upregulation. Our results provide novel insights into the mechanism of CBM 588-mediated anti-inflammatory effects, which can aid in the development of probiotic treatments.

## Data Availability Statement

The metabolomics data generated during the current study were submitted to the EMBL-EBI MetaboLights database with the identifier MTBLS2087.

## Ethics Statement

The animal study was reviewed and approved by the Aichi Medical University.

## Author Contributions

TA, MH, KO, MT, and HM: conceptualization. TA: methodology, investigation all experiments and writing—original draft. TA, SE, and AF: mass-spectrum assays. KI: flow cytometric analysis. TA and MH: formal analysis. HM: funding acquisition and resources. MT and HM: supervision. All authors contributed to the article and approved the submitted version.

## Conflict of Interest

TA, SE, AF, KO, and MT were employees of Miyarisan Pharmaceutical Co., Ltd. and may have conflict of interest. The remaining authors declare that the research was conducted in the absence of any commercial or financial relationships that could be construed as a potential conflict of interest.

## Footnotes

1 https://www.ebi.ac.uk/metabolights/MTBLS2087
