# Supplementary figures and images for: Clostridium butyricum MIYAIRI 588-Induced Protectin D1 Has an Anti-inflammatory Effect on Antibiotic-Induced Intestinal Disorder
Source: Front Microbiol. 2020 Oct 30;11:587725. doi: 10.3389/fmicb.2020.587725 (PMC7661741; doi:10.3389/fmicb.2020.587725)

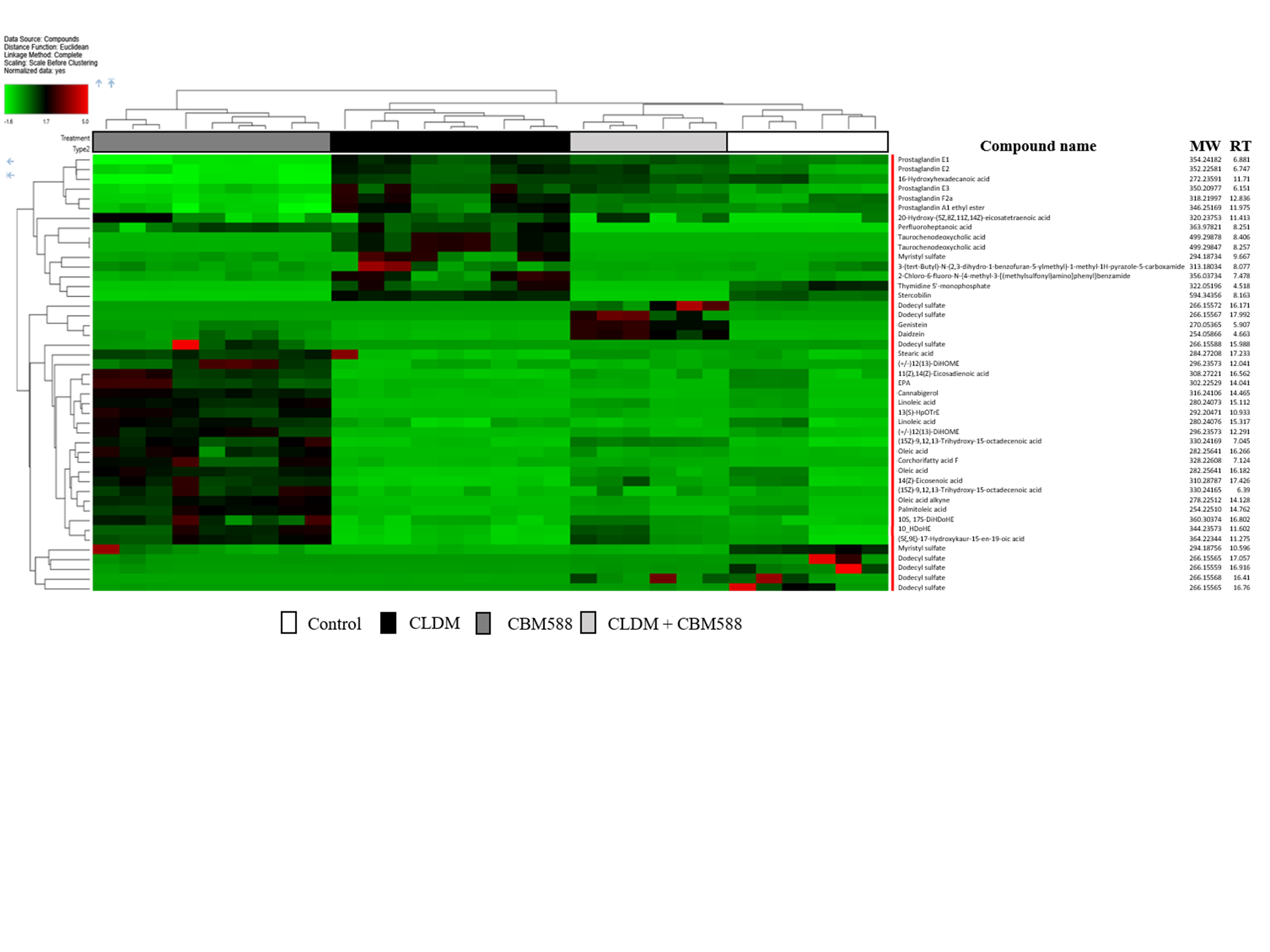

Supplement: Supplementary file 1 [file Image_1.TIF]
