# Supplementary material for: Clostridium butyricum MIYAIRI 588-Induced Protectin D1 Has an Anti-inflammatory Effect on Antibiotic-Induced Intestinal Disorder
Source: Front Microbiol. 2020 Oct 30;11:587725. doi: 10.3389/fmicb.2020.587725 (PMC7661741; doi:10.3389/fmicb.2020.587725)
Supplement: Supplementary file 2 [file Data_Sheet_1.PDF]

*Supplementary Material*

**Supplemental table 1. Linear gradient condition.**

| Min | A (%) | B (%) |
| --- | --- | --- |
| 0 | 80 | 20 |
| 0.5 | 80 | 20 |
| 15 | 2 | 98 |
| 25 | 2 | 98 |
| 25 | 80 | 20 |
| 32 | 80 | 20 |

**Supplemental table 2. Full MS and data-dependent MS/MS method.**

| Full MS | Condition |
| --- | --- |
| Resolution | 7.00E+4 |
| Scan range (*m/z*) | 215-800 |
| AGC target | 1.00E+6 |
| Maximum IT (ms) | 100 |
| Data-dependent MS/MS | Condition |
| Resolution | 1.75E+4 |
| Isolation window (*m/z*) | ±0.8 Da |

| | |
|---|---|
| Stepped normalized collision energy | Step20/40/60 |
| Loop count | 3 |
| AGC target | 5.00E+4 |
| Maximum IT (ms) | 60 |
| Dynamic exclusion | 4 s, 10 s, 30 s |
| Intensity threshold | 8.00E+4 |

**Supplemental table 3. Ionization method.**

| Parameter | Condition |
|---|---|
| Ionization | ESI-Negative |
| Spray voltage (kV) | 3 |
| Vaporizer temperature (°C) | 350 |
| Capillary temperature (°C) | 275 |
| $N_2$ gas flow rate (Sheath gas) | 50 |
| $N_2$ gas flow rate (Aux gas) | 15 |
| S-lens level | 50 |
| Probe position | C, +1.75 |

**Supplemental table 4. Primers used for quantitative real-time RT-PCR.**

| Name | Sequence (5’→3’) | Reference |
|---|---|---|
| GPR41 Forword | GGGGTCGATACAAGAGT | Veprik *et al.*, 2016 |
| GPR41 Reverse | CTGGCGGAGCTACGTGCT | Veprik *et al.*, 2016 |
| GPR43 Forword | CACGGCCTACATCCTCATCT | Fujiwara *et al.*, 2018 |
| GPR43 Reverse | TTGGTAGGTACCAGCGGAAG | Fujiwara *et al.*, 2018 |
| GPR84 Forword | GACTGCCCCTCAAAAGACCTGC | Widmayer *et al.*, 2017 |
| GPR84 Reverse | GCCACGCCCCAGATAATTGC | Widmayer *et al.*, 2017 |
| GPR120 Forword | GTGCCGGGACTGGTCATTGTG | Widmayer *et al.*, 2017 |
| GPR120 Reverse | TTGTTGGGACACTCGGATCTGG | Widmayer *et al.*, 2017 |
| COX-1 Forword | ATGAGTCGAAGGAGTCTCTCG | Hamabata *et al.*, 2018 |
| COX-1 Reverse | GCACGGATAGTAACAACAGGGA | Hamabata *et al.*, 2018 |
| COX-2 Forword | AAGCCGAGCACCTTTGGAG | Hamabata *et al.*, 2018 |
| COX-2 Reverse | ATTGATGGTGGCTGTTTTGGTAG | Hamabata *et al.*, 2018 |
| 5-LOX Forword | ACCAAACCCCTGGAGAGAGTA | Hamabata *et al.*, 2018 |
| 5-LOX Reverse | GCGATACCAAACACCTCAGAC | Hamabata *et al.*, 2018 |
| 15-LOX Forword | CTCTCAAGGCCTGTTCAGGA | Hamabata *et al.*, 2018 |
| 15-LOX Reverse | AGGATTGTGCCATCCTTCCA | Hamabata *et al.*, 2018 |
| IL-1β Forword | TAGACAACTGCACTACAGGCTCCGA | Hagihara *et al.*, 2020 |
| IL-1β Reverse | GGGTCCGACAGCACGAGGCT | Hagihara *et al.*, 2020 |
| TNF-α Forword | TGGTGACCAGGCTGTCGCTACA | Hagihara *et al.*, 2020 |

| | | |
|---|---|---|
| TNF-α Reverse | TACAGTCACGGCTCCCGTGGG | Hagihara *et al.*, 2020 |
| TGF-$\beta_1$ Forword | GTGCCTATGTCTCAGCCTCTT | Hagihara *et al.*, 2020 |
| TGF-$\beta_1$ Reverse | ATTTGGGAACTTCTCATCCCT | Hagihara *et al.*, 2020 |
| IL-10 Forword | CAACCCAAGTAACCCTTAAAGT | Hagihara *et al.*, 2020 |
| IL-10 Reverse | AGCTGCGGACTGCCTTC | Hagihara *et al.*, 2020 |
| IL-4 Forword | CTCATGGAGCTGCAGAGACTCTT | Hagihara *et al.*, 2020 |
| IL-4 Reverse | CATTCATGGTGCAGCTTATCGA | Hagihara *et al.*, 2020 |
| IL-17A Forword | ACTCTCCACCGCAATGAAG | Hagihara *et al.*, 2020 |
| IL-17A Reverse | TTCAGGACCAGGATCTCTTG | Hagihara *et al.*, 2020 |
| CuZnSOD Forword | GGCGTCATTCACTTCGAGCAGAAG | Goto *et al.*, 2002 |
| CuZnSOD Reverse | GGCAATCCCAATCACACCACAAGC | Goto *et al.*, 2002 |
| Gclm Forword | TGGAGCAGCTGTATCAGTGG | Dirscherl *et al.*, 2010 |
| Gclm Reverse | AAATCTGGTGGCATCACACA | Dirscherl *et al.*, 2010 |

**Supplemental Figure 1.** Heatmaps of selected colon lipid metabolites, generated by hierarchical clustering, were significant between the control, CLDM clindamycin, and/or CBM 588 administration groups by One-way analysis of variance (ANOVA) with the Tukey test. $p$ value < 0.05 was considered statistically significant.
